# Current Practices in Data Analysis Procedures in Psychology: What Has Changed?

**DOI:** 10.3389/fpsyg.2018.02558

**Published:** 2018-12-13

**Authors:** María J. Blanca, Rafael Alarcón, Roser Bono

**Affiliations:** ^1^Department of Psychobiology and Behavioral Sciences Methodology, University of Málaga, Málaga, Spain; ^2^Department of Social Psychology and Quantitative Psychology, Faculty of Psychology, University of Barcelona, Barcelona, Spain; ^3^Institute of Neurosciences, University of Barcelona, Barcelona, Spain

**Keywords:** data analysis procedures, empirical research, quantitative research, methodological review, ANOVA, regression analysis

## Abstract

This paper analyzes current practices in psychology in the use of research methods and data analysis procedures (DAP) and aims to determine whether researchers are now using more sophisticated and advanced DAP than were employed previously. We reviewed empirical research published recently in prominent journals from the USA and Europe corresponding to the main psychological categories of Journal Citation Reports and examined research methods, number of studies, number and type of DAP, and statistical package. The 288 papers reviewed used 663 different DAP. Experimental and correlational studies were the most prevalent, depending on the specific field of psychology. Two-thirds of the papers reported a single study, although those in journals with an experimental focus typically described more. The papers mainly used parametric tests for comparison and statistical techniques for analyzing relationships among variables. Regarding the former, the most frequently used procedure was ANOVA, with mixed factorial ANOVA being the most prevalent. A decline in the use of non-parametric analysis was observed in relation to previous research. Relationships among variables were most commonly examined using regression models, with hierarchical regression and mediation analysis being the most prevalent procedures. There was also a decline in the use of stepwise regression and an increase in the use of structural equation modeling, confirmatory factor analysis, and hierarchical linear modeling. Overall, the results show that recent empirical studies published in journals belonging to the main areas of psychology are employing more varied and advanced statistical techniques of greater computational complexity.

## Introduction

In order to answer a specific research question, researchers have to make important decisions about the design and the data analysis procedures (DAP) they will use. Planning and conducting a study is like doing a puzzle, in which all the pieces must be correctly fitted together. Within a given research design there is usually more than one statistical technique that might be used to address the research question, and thus researchers must select a DAP based on the characteristics of their data. Some DAP have to satisfy a number of underlying assumptions, and if these assumptions are not fulfilled the interpretation of results might not be valid.

Data analysis procedures are constantly evolving and new and more sophisticated approaches are frequently being developed and published, thanks partially to the accessibility of powerful and specialized computer programs for statistical data analysis. However, it may take time for new procedures to be implemented in practice and for reports of their use by researchers and practitioners to be published. In this respect, reviews of methodological research can help to describe current practices in the use of DAP and to determine the degree to which new procedures are being used.

Numerous papers have discussed statistical and research practices in several spheres, including medicine (Kuroki et al., 2009; Casals et al., 2014; Otwombe et al., 2014; Brophy et al., 2015; Akhtar et al., 2016), health services (Wisdom et al., 2012), nursing (Gaskin and Happell, 2014), manufacturing strategies (Chatha et al., 2015), management (Ketchen et al., 2008; Asare et al., 2013), second language learning (Plonsky and Gass, 2011; Plonsky, 2013, 2014), education (Willson, 1980; Goodwin and Goodwin, 1985a,b; Elmore and Woehlke, 1988, 1996, 1998; Hsu, 2005; Zientek et al., 2008; Warne et al., 2012), psychology (Edgington, 1964, 1974; Kruglanski, 1975; Reis and Stiller, 1992; West et al., 1992; Baumberger and Bangert, 1996; Schinka et al., 1997; Bangert and Baumberger, 2005; Kashy et al., 2009; Harlow et al., 2013; Fernández-García et al., 2014; Counsell and Harlow, 2017), and psychology and education jointly (Keselman et al., 1998; Kieffer et al., 2001; Skidmore and Thompson, 2010).

The majority of studies conducted in relation to psychology analyze the prevalence of DAP, although there are also some which focus on the use of specific research designs, for example, the quasi-experimental approach (Fernández-García et al., 2014) or statistical data analysis such as ANOVA, ANCOVA, and MANOVA (Keselman et al., 1998), multivariate analysis (Harlow et al., 2013), Bayesian analysis (Van de Schoot et al., 2017), and confirmatory factor analysis (Jackson et al., 2009). Among those studies which analyze the prevalence of DAP, the majority are focused on journals whose aim and scope is to publish articles about specific areas of psychological research, such as personality and social psychology (Kruglanski, 1975; Reynolds and Clark, 1984; Reis and Stiller, 1992; West et al., 1992; Schinka et al., 1997; Kashy et al., 2009), learning disabilities (Baumberger and Bangert, 1996), or counseling and development (Bangert and Baumberger, 2005). By contrast, few studies have covered all areas of psychology in order to provide an overall view of the statistical techniques most frequently used in this discipline.

Edgington (1964, 1974) was one of the first authors to study the prevalence of DAP for all areas of psychological research, albeit restricted to APA journals. He found that by 1972, ANOVA was the procedure used in 71% of articles involving statistical inference. Reis and Stiller (1992) examined research trends in the *Journal of Personality and Social Psychology* and found that in 1988 ANOVA remained by far the most popular technique (even though its prevalence had declined), followed by correlation, multiple regression analysis, and factor analysis. Kieffer et al. (2001) likewise reported that ANOVA and correlation analysis were the most prevalent DAP used in *Journal of Counseling Psychology* articles from 1988 to 1997, a finding consistent with that of Schinka et al. (1997) in their review of articles published in the *Journal of Personality Assessment* from 1990 to 1994. Skidmore and Thompson (2010) conducted a metasynthesis of five articles reviewing the psychological literature from 1948 to 2001 and found that ANOVA techniques began to be less widely used in the 1990s, whereas the use of correlational and regression techniques appeared to be increasing. However, Kashy et al. (2009) found that in 2007, ANOVA remained the most common analytic technique used (52%) in the *Personality and Social Psychology Bulletin*, followed by multiple regression (41%). Consistent with these findings, Counsell and Harlow (2017) reported that 41.2% of the analyses included in four major Canadian psychology journals performed a univariate mean comparison, with ANOVA being the most often reported (24.8%), and 35% of them included techniques for examining associations among variables, with multiple regression being the most prevalent (13.7%).

The above studies have provided useful information about research trends and suggest that the use of DAP has changed little over time: ANOVA, correlation, and multiple regression analysis remain the most frequently used techniques since early research in several areas of psychology. However, there are two reasons why this may not be an accurate reflection of current research. First, most studies that have examined this question are limited to a specific journal and do not cover all areas of psychology, and second, the time frame considered in existing reviews does not provide up-to-date information. The most recent study, that by Counsell and Harlow (2017), considered the prevalence of DAP in articles published in 2013, but the focus was limited to Canadian psychology journals.

The aim of the present paper is to analyze current practices in psychology in the use of research methods and statistical techniques and to determine whether the use of procedures based on more sophisticated mathematical and statistical algorithms (i.e., structural equation modeling, generalized linear mixed models) has increased with respect to the techniques most commonly employed previously (i.e., correlation, ANOVA). In order to provide an overview of the most frequently used DAP in this discipline we examine empirical research published recently in journals belonging to the main psychological categories of Journal Citation Reports (JCR) — namely Applied, Developmental, Experimental, Clinical, Educational, Social, Multidisciplinary, and Psychology — thus enabling us to examine whether or not there are differences between psychological fields. In order to provide knowledge relating to several geographical areas, journals from the USA and Europe are considered. By revealing current practices in DAP this study could help methodologists (1) to identify topics related to statistical data analysis that should be addressed in greater depth, and (2) to reflect critically on the extent to which new and more sophisticated statistical tools are being used and to consider how they can be made more accessible and available to researchers and practitioners. The results should also provide a platform for future studies that analyze aspects related to the quality of published research, focusing especially on those articles which employ the techniques shown here to be the most widely used. By reviewing the application of analytic procedures and the reporting of results these studies could provide best practice guidelines for applied researchers.

## Materials and Methods

### Data Sample

The focus of analysis was scientific journals whose aim and scope is to publish empirical articles in one or more of the main categories of psychological research: Applied, Developmental, Educational, Experimental, Clinical, Social, and Multidisciplinary. The inclusion criteria for journals were: (a) Being indexed by JCR and categorized as Q1 or Q2 in the last 5 years; (b) having a broad scope rather than being restricted to a specific topic in psychology (e.g., abnormal child psychology, autism, perception, etc.); (c) being founded at least 10 years ago; (d) publishing papers written in English; and (e) being classified in only one category in the JCR. The *Psychology* category of JCR was also considered, although none of the journals thus identified met the last inclusion criterion as they were all classified in more than one category (e.g., Psychology and Applied). In this case, we considered those journals which were classified under two categories and met the remaining inclusion criteria. The journals were selected randomly for each category, but making sure that those from the USA and from several geographic areas of Europe were included. The journals selected in each area were:

- *Applied*: Journal of Experimental Psychology: Applied (American Psychological Association, United States).

- *Clinical*: British Journal of Clinical Psychology (the British Psychological Society, United Kingdom).

- *Developmental*: Developmental Psychology (American Psychological Association, United States).

- *Educational*: British Journal of Educational Psychology (the British Psychological Society, United Kingdom).

- *Experimental*: Psychological Research/Psychologische Forschung (Germany).

- *Social*: European Journal of Social Psychology (European Association of Social Psychology, Netherlands).

- *Multidisciplinary*: Psicothema (Colegio Oficial de Psicólogos del Principado de Asturias, Spain).

- *Psychology*: Health Psychology (American Psychological Association, United States).

We examined 36 papers published in 2017 in each of the selected journals. If the total number in a specific journal did not reach this figure (as in the case of the *Journal of Experimental Psychology: Applied* and the *British Journal of Clinical Psychology*), we extended the sampling period, first to 2018 (8 papers included) and, if necessary, to 2016 (3 papers included). If the journal published more than 36 papers in 2017, random articles were selected proportionally from each issue by means of random number generation. A total of 288 articles were reviewed (sample size calculated with a margin of error of 5% and a 97.5% confidence level, based on the total number of articles published in 2017 in the eight journals selected), all of which reported empirical quantitative research (see Supplementary Material). Publications corresponding to editorials, comments, book reviews, errata, theoretical or literature reviews, synthesis of previous research (e.g., meta-analysis, systematic reviews), qualitative research, analytical or mathematical procedures, statistical simulations, tutorials, illustrations, teaching tools, neuroimaging techniques, or software presentations were excluded. Data are available upon request from the authors.

### Measures

Evaluators recorded the following variables:

(1) Paper identification: journal title, JCR category, year, volume and issue number, first and last page numbers.

(2) Research method identification: type of research method(s) used to address the main research question. Research methods were classified as intervention or non-intervention studies (Bangert and Baumberger, 2005), as follows:

Intervention studies, where the researcher manipulates the independent variables in order to establish cause-effect relationship.

- Experimental: the study includes independent variables manipulated by the researcher(s) and random assignment in a between-group design or a random treatment point in a repeated measures design.

- Quasi-experimental: the study includes independent variables manipulated by the researcher but with non-random assignment or a non-random treatment point.

Non-intervention studies, where the researcher does not introduce manipulation in order to describe the sample or to establish an association between variables.

- Descriptive: the aim is to describe the sample without statistical inference.

- Observational: the systematic observation of behavior, including quantitative analysis of the recorded data.

- Survey: the research involves asking respondents questions with the aim of describing or explaining their responses.

- Epidemiology: the study of the occurrence, distribution, and determinants of events related to health, states, and processes in specified populations, and the application of this knowledge to control relevant health problems.

- Correlational/Predictive: the study of the association between variables or the identification of the variables which contribute to the prediction of another variable. Studies whose aim was to estimate growth over a period of time were also included in this category.

- Comparative: the aim is the comparison of pre-existing groups. The grouping variable may be a characteristic of the subjects themselves.

- Cross–cultural: the study of behavior under diverse cultural conditions.

- Classification: although it is not considered as a research method, we included under this category those studies whose aim was to group a set of similar subjects or to identify groups as a function of a data set.

- Psychometric/Measurement: studies involving the analysis of psychometric properties of instruments for measuring psychological variables (e.g., factor structure, validity, reliability, etc.).

(3) Study identification: number of studies reported in each paper, based on separate headings and multiple method sections. Studies or experiments labeled as “a”, “b”, etc. (e.g., Experiment 1a, 1b…) were considered as a single entity.

(4) Data analysis procedure identification: number of different types of DAP performed and type of DAP performed. Here we drew up a wide-ranging list of DAP categories that was reviewed by other experts in statistical analysis. Table 4 shows the categories found in this methodological review.

(5) Statistical package identification: statistical packages used for each DAP. Table 8 shows the categories of statistical package found in the papers examined.

### Procedure

The papers were coded by the authors of the present study, all of whom are experienced researchers with knowledge of applied statistics. Specific, detailed, and clear instructions were drawn up for the coding procedure. In addition, several training sessions were held to refine these instructions and to familiarize ourselves with the coding process. In these sessions, several papers were first coded jointly, and then independently. The definitive coding process began once the code book and the code sheet with its instructions were clear and the three evaluators agreed as to its application.

The first author downloaded electronic copies of the selected papers and recorded the abovementioned variables for each one. In order to assess the reliability of coding, we randomly split the papers from each journal into two half sets, and the second and third authors independently evaluated one half. Any doubts or disagreements about a given DAP, research method, or software package were discussed in the research team.

We recorded a single research method for each paper, unless it included several studies using different methods (this was the case in six papers). If a paper presented multiple studies we recorded each study and each type of different DAP reported. Consequently, the number of DAP exceeded the number of papers reviewed. However, each DAP was counted only once if it was used more than once in the same paper. Each DAP was recorded in a separate row.

We recorded descriptive statistics or correlations only if they were performed to answer a specific research question linked to the aim of the paper. Likewise, statistics reported in the ‘Participants’ section, ‘Measures’ section, or footnotes were not considered, and neither were those related to sections labeled as ‘preliminary results’ or ‘manipulation checking.’

We calculated the percentages associated with the research methods used and number of studies, number and type of DAPs, and statistical analysis software. This information provides an overview of current practices in DAPs in psychology.

## Results

### Research Methods and Number of Studies

Table 1 shows the frequency with which different research methods were used in the papers reviewed. Inter-coder agreement was 99.2% (between the first and second author) and 96.4% (between the first and third author). The most frequently used research methods were experimental (39.46%) and correlational (39.12%). Experimental research was more prevalent in *Psychological Research* (97.22%), the *Journal of Experimental Psychology: Applied* (92.31%), and the *European Journal of Social Psychology* (53.85%), whereas correlational studies were more frequent in *Health Psychology* (80.55%), *Developmental Psychology* (66.67%), the *British Journal of Educational Psychology* (58.33%), and the *British Journal of Clinical Psychology* (44.44%). Psychometric and correlational studies were the most common in *Psicothema* (36.10 and 30.56%, respectively).

**Table 1:** Frequency and percentage of the research methods used in the papers, by journal.

		Percentage							
							*European*
			*British Journal*		*British Journal*		*Journal of*
Research		JEP:	*of Clinical*	Developmental	*of Educational*	Psychological	*Social*		Health
methods	*N* (%)	Applied	*Psychology*	Psychology	*Psychology*	Research	*Psychology*	Psicothema	Psychology
Intervention studies	141 (47.96)	92.31	33.34	30.55	27.77	97.22	58.98	19.45	19.45
Experimental	116 (39.46)	92.31	5.56	22.22	19.44	97.22	53.85	16.67	2.78
Quasi-experimental	25 (8.50)	–	27.78	8.33	8.33	–	5.13	2.78	16.67
Non-intervention studies	153 (52.04)	7.69	66.66	69.45	72.23	2.78	41.02	80.55	80.55
Observational	2 (0.68)	5.13	–	–	–	–	–	–	–
Survey	1 (0.34)	–	–	–	–	–	2.56	–	–
Epidemiology	1 (0.34)	–	2.78	–	–	–	–	–	–
Correlational/Predictive	115 (39.12)	2.56	44.44	66.67	58.33	2.78	30.77	30.56	80.55
Comparative study	13 (4.42)	–	19.44	–	2.78	–	2.56	11.11	–
Cross-Cultural study	2 (0.68)	–	–	2.78	–	–	2.56	–	–
Classification	1 (0.34)	–	–	–	–	–	–	2.78	–
Psychometric/	18 (6.12)	–	–	–	11.12	–	2.57	36.10	–
Measurement
*N* Total	294	39	36	36	36	36	39	36	36

Table 2 shows the distribution of the number of studies or experiments included in the articles reviewed: this number ranged from 1 to 5 (overall mean of 1.56), with 69.44% of papers reporting a single study. The *Journal of Experimental Psychology: Applied*, *Psychological Research*, and the *European Journal of Social Psychology* were the journals in which individual papers reported a higher number of studies.

**Table 2:** Frequency, percentage, mean (M), and standard deviation (SD) for the number of studies or experiments included in the papers, by journal.

		Percentage							
							*European*
			*British Journal*		*British Journal*		*Journal of*
Number of		JEP:	*of Clinical*	Developmental	*of Educational*	Psychological	*Social*		Health
studies	*N* (%)	Applied	*Psychology*	Psychology	*Psychology*	Research	*Psychology*	Psicothema	Psychology
1	200 (69.44)	16.67	97.22	86.11	94.44	33.33	30.56	97.22	100
2	39 (13.54)	27.78	2.78	13.89	5.56	30.56	25.00	2.78	–
3	30 (10.42)	25.00	–	–	–	25.00	33.33	–	–
4	15 (5.21)	19.44	–	–	–	11.11	11.11	–	–
5	4 (1.39)	11.11	–	–	–	–	–	–	–
*M*	1.56	2.81	1.03	1.14	1.06	2.14	2.25	1.03	1
*SD*	0.97	1.26	0.17	0.35	0.23	1.02	1.03	0.17	–
*N* Total	288	36	36	36	36	36	36	36	36

### Number and Type of Data Analysis Procedures

The 288 papers used 663 different DAP. The inter-coder agreement was 94.6% (between the first and second author) and 96.5% (between the first and third author). Table 3 shows the number of different DAP performed. This ranged from 1 to 8 per article (overall mean of 2.31), with 60.07% of papers performing more than one type of analysis.

**Table 3:** Frequency, percentage, mean (M), and standard deviation (SD) for the number of data analysis procedures (DAP) used in the papers, by journal.

		Percentage							
							*European*
			*British Journal*		*British Journal*		*Journal of*
Number of		JEP:	*of Clinical*	Developmental	*of Educational*	Psychological	Social		Health
DAP	*N* (%)	Applied	*Psychology*	Psychology	*Psychology*	Research	*Psychology*	Psicothema	Psychology
1	115 (39.93)	22.22	36.11	47.22	52.78	55.56	25.00	13.89	66.67
2	70 (24.31)	30.56	27.78	27.78	19.44	30.56	16.67	22.22	19.44
3	51 (17.71)	19.44	8.33	13.89	25.00	8.33	33.33	22.22	11.11
4	27 (9.38)	8.33	19.44	11.11	–	5.56	11.11	16.67	2.78
5	12 (4.17)	11.11	5.56	–	–	–	8.33	8.33	–
6	6 (2.08)	–	2.78	–	–	–	2.78	11.11	–
7	4 (1.39)	5.56	–	–	–	–	–	5.56	–
8	3 (1.04)	2.78	–	–	2.78	–	2.78	–	–
*M*	2.31	2.92	2.39	1.89	1.89	1.64	2.83	3.39	1.50
*SD*	1.52	1.84	1.44	1.04	1.35	0.87	1.61	1.76	0.81
*N* Total	288	36	36	36	36	36	36	36	36

Table 4 shows percentages for the different type of DAP used. It can be seen that the most commonly used procedure was ANOVA (20.81%), followed by regression analysis (12.37%).

**Table 4:** Frequency and percentage of the data analysis procedures (DAP) performed in the papers.

DAP	*N*	Percentage
Descriptive statistics (M, SD, OR, RR, percentages, etc.)	15	2.26
Distribution fitting	3	0.45
Inter-rater agreement (Kappa, Intraclass correlation coefficient)	3	0.45
Measures of association		
Pearson’s correlation coefficient	54	8.14
Pearson’s correlation/regression coefficient comparison test	3	0.45
Spearman’s correlation coefficient	8	1.21
Other nominal/ordinal correlation measures (gamma, Cramer’s *V*, Somers’ *d*, contingency coefficient)	4	0.60
Tests of contingency tables and for proportion comparison		
Pearson’s Chi-square	27	4.07
Other test of contingency tables and proportion comparison (Fisher, McNemar tests, Cochran’s *Q*, z statistic)	5	0.75
Non-parametric tests for comparison		
Mann–Whitney *U* test	5	0.75
Wilcoxon signed-rank test	4	0.60
Kruskal–Wallis test	5	0.75
Friedman test	5	0.75
Parametric tests for comparison		
One sample *t*-test	11	1.66
Independent *t*-test	45	6.79
Paired *t*-test	20	3.02
ANOVA	138	20.81
ANCOVA	15	2.26
MANOVA/MANCOVA	13	1.96
Regression models to analyze relationships among variables		
Regression analysis	82	12.37
Multilevel regression	25	3.77
Multivariate regression	1	0.15
Poisson regression	1	0.15
Logistic regression	15	2.26
Multilevel logistic regression	5	0.75
Multinomial/ordinal regression	9	1.36
Generalized estimation equation for ordinal data	1	0.15
Structural equation modeling (SEM) to analyze relationships among variables		
Path analysis	46	6.94
Multilevel SEM	6	0.90
Growth curve modeling	10	1.51
Multilevel growth curve modeling	2	0.30
Psychometric: Factor analysis		
Confirmatory factor analysis (CFA, SEM)	21	3.17
Exploratory factor analysis (EFA)	13	1.96
Other DAP for psychometric analysis
Cronbach’s alpha	17	2.56
McDonald’s omega	4	0.60
Test–retest reliability	3	0.45
Convergent/Discriminant validity indexes (AVE, MSV)	2	0.30
Sensitivity and specificity measures	2	0.30
Item analysis: Classical test theory	2	0.30
Item analysis: Item response theory (item calibration, DIF)	2	0.30
Other analyses		.
Cluster analysis	3	0.45
ROC curve analysis	7	1.06
Markov models	1	0.15
Total	663	

Table 5 groups the different types of DAP into broader categories and shows their frequency of use by journal. The most widely used procedures were parametric tests for comparison (36.50%) and regression models to analyze relationships between variables (20.97%). It can be seen that papers published in *Psychological Research* mainly used parametric tests for comparison (83.05%), whereas papers in *Health Psychology* were more likely to involve the analysis of relationships among variables using either regression models (51.85%) or structural equation modeling (SEM) (22.22%). Parametric tests for comparison were also the most prevalent in the *Journal of Experimental Psychology: Applied* (50.48%) and the *European Journal of Social Psychology* (42.57%), and models for analyzing relationships (both regression and SEM) were the most common in *Developmental Psychology* and the *British Journal of Educational Psychology* (51.47% and 44.12%, respectively). The DAP used in the *British Journal of Clinical Psychology* and *Psicothema* were more varied.

**Table 5:** Frequency and percentage of the data analysis procedures (DAP) performed in the papers, by journal.

		Percentage							
							*European*
			*British Journal*		*British Journal*		*Journal of*
		JEP:	*of Clinical*	Developmental	*of Educational*	Psychological	*Social*		Health
DAP	*N* (%)	Applied	*Psychology*	Psychology	*Psychology*	Research	*Psychology*	Psicothema	Psychology
Descriptive statistics	15 (2.26)	2.86	6.98	–	1.47	1.69	0.99	2.46	–
Distribution fitting	3 (0.45)	0.95	–	–	–	–	–	0.82	1.85
Inter-rater agreement	3 (0.45)	2.86	–	–	–	–	–	–	–
Measures of association	69 (10.41)	10.48	9.30	5.88	11.76	6.78	9.90	17.21	5.56
Tests of contingency tables and for proportion comparison	32 (4.83)	7.62	5.81	7.35	2.94	–	3.96	3.28	7.41
Non-parametric tests for comparison	19 (2.87)	1.90	9.30	2.94	1.47	1.69	–	4.10	–
Parametric tests for comparison	242 (36.50)	50.48	31.40	27.94	19.12	83.05	42.57	26.23	11.11
Regression models to analyze relationships among variables	139 (20.97)	17.14	19.77	23.53	22.06	6.78	29.70	9.02	51.85
SEM to analyze relationships among variables	64 (9.65)	–	8.14	27.94	22.06	–	6.93	3.28	22.22
Psychometric: Factor analysis	34 (5.13)	0.95	2.33	4.41	10.29	–	2.97	14.75	–
Other DAP for psychometric analysis	32 (4.83)	1.90	4.65	–	7.35	–	2.97	14.75	–
Other analyses	11 (1.66)	2.86	2.33	–	1.47	–	–	4.10	–
*N* Total	663	105	86	68	68	59	101	122	54

In Table 6 the same broader categories of DAP are considered in terms of their proportional use within the context of the different research methods. Obviously, parametric tests for comparison were the most prevalent in experimental, cross-cultural, comparative, and quasi-experimental studies (in this order), whereas the use of regression models and SEM to analyze relationships among variables were the most common in correlational research.

**Table 6:** Percentage of the data analysis procedures (DAP) performed in the papers, by type of research method.

		Quasi-				Correlational	Comparative	Cross-		Psychometric
DAP	Experimental	experimental	Observational	Survey	Epidemiology	Predictive	studies	cultural	Classification	Measurement
Descriptive statistics	1.52	4.55	20.00	–	50.00	1.84	–	–	–	2.41
Distribution fitting	0.76	–	–	–	–	–	–	–	–	1.20
Inter-rater agreement	0.38	–	40.00	–	–	–	–	–	–	–
Measures of association	9.89	4.55	–	–	–	11.06	8.57	–	–	16.87
Tests of contingency tables and for proportion comparison	5.32	6.82	–	–	–	5.07	5.71	14.29	–	1.20
Non-parametric tests for comparison	1.52	9.09	–	–	25.00	1.84	8.57	–	–	3.61
Parametric tests for comparison	61.60	47.73	20.00	–	–	12.90	51.43	57.14	33.33	8.43
Regression models to analyze relationships among variables	16.35	20.45	–	100	–	35.48	11.43	28.57	–	2.41
SEM to analyze relationships among variables	0.38	6.82	–	–	–	26.27	5.71	–	–	1.20
Psychometric: Factor analysis	0.38	–	–	–	–	3.69	–	–	–	30.12
Other DAP for psychometric analysis	0.76	–	–	–	–	1.38	5.71	–	–	30.12
Other analyses	1.14	–	20.00	–	25.00	0.46	2.86	–	66.67	2.41

In order to determine which specific procedure was more commonly used in the two most popular types of analysis (i.e., ANOVA and regression analysis) we re-examined the papers using more refined categories for these two approaches. This analysis showed that the types of ANOVA used were: Between-subjects one-way ANOVA, repeated measures one-way ANOVA, between-factors ANOVA, repeated measures factorial ANOVA, mixed factorial ANOVA, and a Bayesian approach to ANOVA. For regression analysis the specific procedures used were: Simple linear regression, multiple linear regression (“enter” method), stepwise/backward regression, hierarchical regression, modeling approach to multiple regression, and mediation analysis with regression. These results are shown in Table 7. Since each of these categories corresponded to a different type of DAP, the total number of procedures based on ANOVA or regression analysis exceeds the number reported in Table 4 (where all ANOVA or regression categories were counted only once). It can been seen in Table 7 that mixed factorial ANOVA was the most common type of this approach (36.61%), while in the case of regression the most frequently used procedures were mediation analysis (32.65%) and hierarchical regression (31.63%).

**Table 7:** Frequency and percentage of the data analysis procedures (DAP) corresponding to ANOVA and regression analysis, by journal.

	Percentage								
DAP	*N* (%)	JEP: Applied	*British Journal of Clinical Psychology*	Developmental Psychology	*British Journal of Educational Psychology*	Psychological Research	*European Journal of Social Psychology*	Psicothema	Health Psychology
**ANOVA**									
Between-subjects one-way ANOVA	45 (24.59)	22.45	41.18	–	28.57	2.22	31.03	63.16	60.00
Repeated measures one-way ANOVA	9 (4.92)	10.20	5.88	–	–	4.44	3.45	–	–
Between-factors ANOVA	29 (15.85)	12.24	–	16.67	28.57	2.22	51.72	10.53	20.00
Repeated measures factorial ANOVA	30 (16.39)	14.29	–	25.00	–	44.44	–	–	–
Mixed factorial ANOVA	67 (36.61)	36.73	52.94	58.33	42.86	44.44	13.79	26.32	20.00
Bayesian approach to ANOVA	3 (1.64)	4.08	–	–	–	2.22	–	–	–
***N* TOTAL**	183	49	17	12	7	45	29	19	5
**Regression analysis**									
Simple linear regression	4 (4.08)	–	7.14	–	–	33.33	3.85	–	7.69
Multiple linear regression: Enter method	23 (23.47)	21.43	21.43	42.86	30.00	66.67	19.23	27.27	7.69
Stepwise/backward regression	3 (3.06)	7.14	7.14	–	–	–	–	9.09	–
Hierarchical regression	31 (31.63)	21.43	42.86	14.29	50.00	–	23.08	36.36	46.15
Modeling approach to multiple regression	5 (5.10)	7.14	–	–	10.00	–	–	–	23.08
Mediation analysis with regression	32 (32.65)	42.86	21.43	42.86	10.00	–	53.85	27.27	15.38
***N* TOTAL**	98	14	14	7	10	3	26	11	13

### Statistical Analysis Software

For statistical analysis packages the inter-coder agreement was 98.3% (between the first and second author) and 98.1% (between the first and third author). Results are shown in Table 8 as a function of the DAP. The majority of the analyses performed did not identify the software used (55.66%). Of those that did, the most widely used software was IBM SPSS (20.36%), followed by MPLUS, PROCESS Macro, and AMOS. MPLUS was the most commonly used package for analyzing relationships via SEM and factor analysis, followed by AMOS.

**Table 8:** Frequency and percentage for the use of statistical packages according to the data analysis procedure (DAP): (1) Descriptive statistics, (2) distribution fitting, (3) inter-rater agreement, (4) measures of association, (5) tests of contingency tables and for proportion comparison, (6) non-parametric tests for comparison, (7) parametric tests for comparison, (8) regression models to analyze relationships among variables, (9) SEM to analyze relationships among variables, (10) psychometric, factor analysis, (11) other DAP for psychometric analysis, and (12) other analyses.

		Percentage											
Statistical package	*N* (%)	1	2	3	4	5	6	7	8	9	10	11	12
NOT STATED	369 (55.66)	46.67	66.67	100	66.67	65.63	63.16	81.40	38.85	7.81	17.65	28.13	63.64
IBM SPSS	135 (20.36)	53.33	33.33	–	30.43	31.25	36.84	16.12	20.86	–	17.65	31.25	36.36
MPLUS	59 (8.90)	–	–	–	1.45	–	–	0.41	5.76	60.94	26.47	3.13	–
PROCESS MACRO	26 (3.92)	–	–	–	–	–	–	–	18.71	–	–	–	–
AMOS	23 (3.47)	–	–	–	–	–	–	–	–	20.31	20.59	9.38	–
R	11 (1.66)	–	–	–	–	–	–	0.41	1.44	3.13	2.94	15.63	–
STATA	11 (1.66)	–	–	–	–	–	–	0.41	5.76	3.13	–	–	–
HTLM	7 (1.06)	–	–	–	1.45	–	–	–	4.32	–	–	–	–
SAS	9 (1.36)	–	–	–	–	3.13	–	0.83	3.60	1.56	–	–	–
EQS	3 (0.45)	–	–	–	–	–	–	–	–	1.56	5.88	–	–
FACTOR	3 (0.45)	–	–	–	–	–	–	–	–	–	2.94	6.25	–
LISREL	2 (0.30)	–	–	–	–	–	–	–	–	–	5.88	–	–
MLWIN	1 (0.15)	–	–	–	–	–	–	–	.72	–	–	–	–
OTHER SOFTWARE	4 (0.60)	–	–	–	–	–	–	0.41	–	1.56	–	6.25	–
*N* Total	663	15	3	3	69	32	19	242	139	64	34	32	11

## Discussion

The aim of this paper was to analyze current practices in psychology in the use of research methods and statistical techniques and to determine whether researchers are now using more sophisticated and advanced techniques than were employed previously. We reviewed empirical research published recently in prominent journals from the USA and Europe corresponding to the main psychological categories of JCR and examined research methods, the number of studies, the number and type of DAP performed, and the statistical package used for the analysis.

Regarding research methods, the results showed that experimental and correlational studies were the most prevalent in psychological science. Experimental reports were mainly published in the *Journal of Experimental Psychology: Applied* and *Psychological Research*, reflecting the fact that the aim of these journals is to support basic and applied experimental research that is conducted within laboratory or field settings. Around half of the papers from the *European Journal of Social Psychology* also involved experimental research.

Correlational studies were by far the most prevalent in the journal *Health Psychology*, and they also accounted for the largest proportion in *Developmental Psychology* and the *British Journal of Educational Psychology*. Articles published in the *British Journal of Clinical Psychology* featured a wider variety of research methods, mainly correlational, quasi-experimental, and comparative studies. Research published in *Psicothema* also involved a range of methods, which is typical of a multidisciplinary journal, although psychometric and correlational studies were the most prevalent.

Overall, these results suggest that experimental studies are more prevalent in journals associated with the applied, experimental, and social categories of the JCR, whereas the analysis of relationships among variables is a specific aim of publications linked to the health, developmental, educational, and clinical categories. Note also that comparative studies and quasi-experimental research are more common in the clinical field, where research often involves a comparison of pre-existing groups and non-randomized or pretest-posttest designs in order to assess the impact of an intervention. In the journals examined, research involving observation, surveys, epidemiological analysis, cross-cultural studies, and classification was almost non-existent.

In relation to the number of studies reported by individual papers and the number of different DAP used, the results indicated that approximately two-thirds of the articles described a single study. Papers describing two or more studies were typically published in journals with a more experimental focus, such as the *Journal of Experimental Psychology: Applied* or *Psychological Research*. This illustrates how experimental method often involves a sequential approach to a specific research question, with a series of linked experiments being designed in accordance with a specific plan for data collection.

Regarding the number of different DAP performed, the majority of the papers reported more than one analysis; *Psicothema*, the *Journal of Experimental Psychology: Applied*, and the *European Journal of Social Psychology* were the journals in which studies used a greater number of different DAP. These results illustrate how psychological science involves the use of a wide range of statistical techniques, both univariate and multivariate. The most prevalent type of DAP were parametric tests for comparison and regression models for studying predictors and relationships among variables. This is consistent with the fact that the most common research methods are experimental, which mainly uses tests for the comparison of means, and correlational, which relies more on techniques for modeling relationships. More specifically, our results showed that experimental papers and those reporting quasi-experimental, cross-cultural, and comparative studies involved the use of parametric tests for comparison, whereas correlational papers employed procedures based on regression analysis and SEM. The frequency of use of these DAP was also consistent with the methodological focus of the journal in question. For example, papers published in *Psychological Research*, in which 97.22% of reports were experimental, mainly used parametric tests for comparison, whereas articles in *Health Psychology*, in which 80.55% of reports were correlational studies, mostly used statistical models (regression or SEM) to analyze relationships. This suggests that differences in the use of DAP according to the field of psychology may reflect the different research methods used in each field to address a specific research question.

ANOVA was the most widely used procedure, with a prevalence of 20.81%. Since 1956, when the use of ANOVA exceeded that of *t*-tests in several APA journals (Edgington, 1964), ANOVA has remained the most common procedure in the majority of studies published in a variety of psychological journals (e.g., Edgington, 1974; Reis and Stiller, 1992; Schinka et al., 1997; Kieffer et al., 2001; Kashy et al., 2009; Counsell and Harlow, 2017), with a prevalence ranging from 24.8% (Counsell and Harlow, 2017) to 80.2% (Edgington, 1974). Although comparison across studies of this kind is difficult because the journals considered and the procedures for collecting data are different, the percentage of use of ANOVA found here is very similar to that reported by Counsell and Harlow (2017) for several Canadian journals of psychology in 2013.

Among those analyses which included ANOVA, factorial ANOVA was more frequently used than was one-way ANOVA, this being consistent with the findings of Edgington (1974) and, more recently, Golinski and Cribbie (2009). Mixed factorial ANOVA was the most prevalent procedure of this kind. Factorial designs enable a more in-depth analysis of a phenomenon as they allow researchers to study the effect of two or more independent variables. In addition, mixed factorial ANOVA is widely used in laboratory-based psychological experimentation. The main advantage of designs involving repeated measures is that they use experimental units as their own controls, reducing the error variance (Kirk, 2013).

Regression analysis was the second most commonly used DAP, with a prevalence of 12.37%. This is also consistent with previous research (e.g., Reis and Stiller, 1992; Schinka et al., 1997; Kieffer et al., 2001; Kashy et al., 2009; Counsell and Harlow, 2017), in which its prevalence ranged from 8.4% (Reis and Stiller, 1992) to 41% (Kashy et al., 2009). Once again, our figure is very similar to the 13.7% reported by Counsell and Harlow (2017). Harlow et al. (2013), in a review of multivariate analysis in European journals, also found that regression analysis was the most widely used multivariate procedure. In the present study, hierarchical regression and mediation analysis were the most common types of regression analysis.

Other DAP that were frequently used to analyze relationships among variables were: (a) measures of associations, mainly Pearson coefficient, which accounted for 10.41% of the analyses; (b) statistical techniques based on SEM, with a prevalence of 9.65%; (c) multilevel statistical models for testing predictors and associations, with a prevalence of 5.72%; and (d) in the psychometric field, confirmatory factor analysis, with a prevalence of 3.17%.

Regarding statistical packages, the majority of analyses performed did not report the software used. This was particularly the case when more advanced statistical tools were not employed in the analysis. The most widely used software was IBM SPSS (20.36%), for a wide variety of DAP. However, MPLUS and AMOS were more frequently used for SEM and factor analysis. PROCESS Macro was also common as the tool for implementing mediation and moderation analysis in statistical packages such as SAS or SPSS (Hayes, 2017). All mediation analyses performed with this macro used the bootstrapping approach to estimate several effects.

The question that follows from our results is whether or not anything has changed in the use of DAP in psychological science. The answer is yes and no. What has not changed is that ANOVA and regression analysis remain the most popular statistical techniques. However, this does not mean that psychological research is not evolving, but rather reflects the fact that the main research methods used in psychology are experimental and correlational in nature. What matters most is that the DAP used are appropriate and consistent with the research method chosen to address a specific research question, and this is what our results suggest. Parametric tests for comparison are more suitable in experimental, quasi-experimental, cross-cultural, and comparative studies, whereas models for testing predictors and relationships (e.g., regression analysis and SEM) are more suitable for correlational studies. As Wilkinson and the APA Task Force on Statistical Inference (TFSI) have highlighted, although complex analytic methods are sometimes necessary to address research questions effectively, simpler classical approaches can often provide elegant and sufficient answers to important questions (Wilkinson and The Task Force on Statistical Inference, 1999). Nevertheless, this frequent use of ANOVA and regression analysis warrants further investigation with regard to the robustness of these techniques when underlying assumptions are not met, and new statistical procedures might need to be developed for those situations where they are shown not to be robust.

In terms of what has changed, our results also provide information about some of the advances made in psychological science. As noted earlier, comparison across studies of this kind is difficult because of differences in the journals considered, the procedure for collecting data, and the number and categorization of statistical techniques coded. Nonetheless, two main conclusions can be drawn. First, researchers are now using multiple and more varied statistical techniques to answer research questions, thus illustrating how psychological science continues to evolve. Second, the use of basic statistical techniques has become less common and researchers are increasingly employing more advanced and sophisticated procedures. The most important changes are:

- A decline in the use of non-parametric analysis, which accounted for only 2.85% of the analyses. According to Skidmore and Thompson (2010), the use of non-parametric techniques peaked in the 1960s, when they were employed in one-third of analyses, their prevalence decreasing to 18.38% between 1990 and 1997. This change may be due to the kinds of research question that can be addressed using traditional non-parametric procedures such as the Mann–Whitney or Kruskal–Wallis tests. It is also possible that researchers no longer turn to non-parametric techniques when the assumptions of parametric analysis are not met, as is recommended in some classical handbooks on methodology and statistics.

- A decline in the use of automatic predictor selection procedures such as stepwise regression analysis, in which predictors are automatically added or removed from the regression model in steps based on statistical algorithms. Only three papers used this kind of analysis, which accounted for 0.45% of the total DAP and 3.06% of the total regression analyses performed. It is worth noting that Kieffer et al. (2001) reported a prevalence for this technique of 22% in 1996. Given the empirical evidence that stepwise regression is not necessarily able to identify the best predictors (Thompson, 1995), a decline in the use of this procedure suggests an improvement in statistical analysis.

- The choice of hierarchical regression and mediation analysis as preferred techniques for testing relationships among variables. Hierarchical regression involves building several regression models by adding predictors step by step, and allows the researcher to determine whether this addition improves the model. Mediation analysis of psychological processes is useful for theory development and testing, and it allow associations to be decomposed into components that reveal possible causal mechanisms (Shrout and Bolger, 2002). These results indicate that psychological researchers are conducting in-depth studies of phenomena, using multivariate analyses that include multiple predictors, that compare models, and which identify moderator variables and mediating processes.

- An increase in the use of models for identifying relationships among variables via SEM, including growth curve modeling and path analysis. This is in line with the study by Harlow et al. (2013), who found that SEM was the second most widely used multivariate analysis in European journals in 2008. As Skidmore and Thompson (2010) pointed out, the availability of more specialized software for SEM, such as MPLUS, may have made it more accessible to practitioners and, thus, have led to an increased use of these models.

- An increase in the use of multilevel approaches or hierarchical linear modeling. Other authors, such as Counsell and Harlow (2017), reported a prevalence of 2% for 2013. Hierarchical linear modeling assumes that data are hierarchically organized and allows researchers to study the contribution of variables at each level of the hierarchy.

- Confirmatory factor analysis is now more widely used than is exploratory analysis. This implies that researchers are now more frequently using hypothesis-driven modeling based on past evidence and theory, rather than exploring relationships among variables, and this suggests theoretical progress in psychology. Obviously, one would expect the prevalence of confirmatory factor analysis to be higher in methodological reviews that focus on specialized journals in psychological assessment. It might be of interest, therefore, for future studies to examine the practice of statistical analysis specifically in the psychometric field.

This study has shown the extent and nature of DAP used in recent research in prominent American and European journals covering the main areas of psychological research. Designing an exhaustive classification of DAP and coding them has proved time consuming and has implied a considerable research effort. Our study does, however, have certain limitations that need to be acknowledged. First, as we have focused on eight journals with a high impact factor the generalizability of results may be limited. The inclusion of other journals might reveal different practices in relation to DAP, and one task for future research would be to compare these practices among journals with different impact factors. Second, we have studied the use of DAP at a specific point in time (i.e., papers published in 2017) rather than over a longer period. It would therefore be interesting to study the trend over the coming years with similar procedures to those used here in order to enable comparison. Third, we did not consider the quality of the statistical analysis reports. Future studies of this kind should therefore specifically record whether the DAP have been performed and reported correctly, in accordance with APA recommendations (Wilkinson and The Task Force on Statistical Inference, 1999). In fact, some researchers have already sought to address this issue (Jackson et al., 2009; Kashy et al., 2009; Bakker and Wicherts, 2011; Barry et al., 2016; Nuijten et al., 2016; Giofrè et al., 2017).

Overall, the results show that recent empirical studies published in journals belonging to the main areas of psychology are employing more varied and advanced statistical techniques of greater computational complexity. The development of specific and more user-friendly statistical packages may also have contributed to the implementation of these statistical techniques. Despite this improvement in statistical data analysis, it remains the responsibility of methodologists to ensure that the more advanced and sophisticated techniques are accessible to a wide range of researchers and practitioners, who need to be trained in their proper application. In this regard, it would be useful to develop user-friendly guidelines covering the appropriate use of statistical procedures and the reporting of results in order to promote good practices in statistical data analysis and to improve future research. Greater emphasis needs to be placed on techniques for the comparison of means and for analyzing complex interrelationships among variables. Hierarchical linear modeling and analysis based on SEM are becoming more common and their use will likely be further consolidated in the near future.

## Author Contributions

MB, RA, and RB conceived and designed the study, designed the code book, and wrote, reviewed, and edited the manuscript. MB performed the coding, analyzed the data, and drafted the manuscript. RA and RB responsible for the reliability of coding.

## Conflict of Interest Statement

The authors declare that the research was conducted in the absence of any commercial or financial relationships that could be construed as a potential conflict of interest.
